# Detecting relevant changes and responsiveness of Neck Pain and Disability Scale and Neck Disability Index

**DOI:** 10.1007/s00586-012-2407-8

**Published:** 2012-07-03

**Authors:** Wim Jorritsma, Pieter U. Dijkstra, Grietje E. de Vries, Jan H. B. Geertzen, Michiel F. Reneman

**Affiliations:** 1 Department of Rehabilitation Medicine, Center for Rehabilitation, University Medical Center Groningen, University of Groningen, PO Box 30.002, 9750 RA Haren, Groningen, The Netherlands; 2 Department of Oral and Maxillofacial Surgery, University Medical Center Groningen, University of Groningen, Groningen, The Netherlands; 3 Department of Pulmonary Medicine and Tuberculosis, University Medical Center Groningen, University of Groningen, Groningen, The Netherlands

**Keywords:** Clinically important change, Neck disability questionnaires, Non-specific neck pain, Disability

## Abstract

**Purpose:**

To investigate relevant change on the Neck Pain and Disability Scale (NPAD) and Neck Disability Index (NDI) and which questionnaire is the most responsive in patients with non-specific chronic neck pain (CNP).

**Methods:**

Seventy-six patients with non-specific CNP in an outpatient tertiary rehabilitation setting were dichotomized into “improved” and “stable” based on global perceived effect (GPE) scores. To investigate relevant change minimal detectable change (MDC) and minimal important change (MIC) with the receiver operator characteristic (ROC) cut-off point were assessed. Comparison of responsiveness was performed using areas under the ROC curve (AUC) and correlations between change scores of NPAD and NDI, and GPE.

**Results:**

MDC and MIC on NPAD (scale 0–100) were 31.7 and 11.5 points, respectively. MDC and MIC on NDI (scale 0–50) were 8.4 and 3.5 points, respectively. Changes should exceed this MDC or MIC cut-off to be interpreted as relevant. AUC was 0.75 for both NPAD and NDI. Correlations between change scores of NPAD and NDI, and GPE were, respectively, 0.48 (95 % CI 0.29–0.64) and 0.49 (95 % CI 0.30–0.64).

**Conclusions:**

Relevant change on both NPAD and NDI assessed with MDC and MIC resulted in different cut-offs and consequently with different amounts of certainty that the patient is improved. Responsiveness of NPAD and NDI was similar.

## Introduction

The most frequently used neck disability questionnaires are the Neck Pain and Disability Scale (NPAD) [37] and Neck Disability Index (NDI) [35], which are validated in several languages [4, 16, 21, 22, 38]. To evaluate the effect of treatment programs for neck disorders it is necessary that questionnaires are responsive, i.e., have the ability to detect clinical important changes over time. There is a need to define minimum changes in scores on questionnaires that are relevant from patients-, clinicians- or socioeconomic perspectives [34]. To determine relevant change two concepts of interpretability are described [1, 3, 8, 10, 34]. In a distribution-based approach the statistical characteristics of the sample are used to express the observed change in a standardized metric [8, 10, 34]. The most commonly used measure is the minimal detectable change (MDC) [3, 5–7, 9, 10, 16, 22, 29, 32, 36, 38, 40]. The MDC assesses the minimal magnitude of change required to be confident that the observed change reflects ‘real’ change and not measurement error [1, 8, 10, 30, 34]. A major limitation of distribution-based approaches is that they are statistical measures which by themselves do not provide a good indication of the clinical relevance of the observed change [8, 10, 34].

The anchor-based approach assesses which change on a questionnaire corresponds with an important change defined on an external criterion or anchor [for example global perceived effect (GPE)] [8, 10, 17, 34]. The most common method in this approach is the calculation of the minimal important change (MIC) determined by the receiver operator characteristic (ROC) curve cut-off point [6–8, 10, 23, 29, 33, 34, 40]. A major limitation of the anchor-based approach is the absence of a gold standard for the external criterion. A further limitation is that it does not take measurement precision into account and therefore does not necessarily imply statistical significance [8, 10, 34]. Hence, studies which apply both approaches are relevant for clinicians and researchers [8, 10, 34]. Moreover, there is a need of studies that assess relevant changes and compare the responsiveness of neck disability questionnaires applied at the same time to the same sample of patients using the same methods to investigate which questionnaire is most appropriate [28]. There are no studies assessing the MDC and the MIC as concepts of interpretability of relevant change for both NPAD and NDI. The aim of this study was to investigate relevant change on the NPAD and NDI and to investigate which questionnaire is most responsive in a single sample of patients with non-specific chronic neck pain (CNP) in an outpatient tertiary rehabilitation setting.

## Materials and methods

### Study sample

Patients with CNP were recruited from referrals from general practitioners or medical specialists for diagnostic procedures as well as advices and rehabilitation treatment in a tertiary university center for rehabilitation in the Netherlands. To be admitted for a multidisciplinary pain rehabilitation, patients had to agree with the time-contingent approach to restore activities and to facilitate return to work. Inclusion criteria for this study were non-specific CNP (>3 months duration), admitted for outpatient rehabilitation, age between 18 and 65 years, and sufficient knowledge of the Dutch language to complete questionnaires. Neck pain was labeled as “non-specific” or mechanical when the neck pain was produced or aggravated by neck movements or sustained neck postures and no specific underlying pathology could be established [2, 13]. Exclusion criteria were status post neck surgery, co-morbidity severely diminishing physical or mental capacity, pregnancy, addiction to drugs, and extensive psychological or behavioral problems. Specific neck pain and exclusion criteria were assessed based on clinical examination with help of “red flags” and “orange flags” and based on the information of the referrals [25, 26, 31].

### Procedures

Prior to the first visit (*T*_0_) a questionnaire to assess patient and clinical characteristics was filled out. During *T*_0_ a review of the medical history and a physical examination was performed. A second visit (*T*_1_) was scheduled, prior to the start of the multidisciplinary rehabilitation program. During *T*_1_ the patients filled out the NPAD and NDI. After completion of the program varying from 3 to 5 months (*T*_2_), patients filled out the NPAD, NDI, and the GPE. All patients signed informed consent for their data to be used for research. Data were gathered as part of care as usual between November 2006 and October 2010.

### Measurements

The NPAD consists of 20 items [37]. Each item has a VAS of 100 mm with numeric anchors at 0, 1, 2, 3, 4, and 5 (each 20 mm apart). Item scores range from 0 (no pain or activity limitation) to 5 (as much pain as possible or maximal limitation). The total NPAD score ranges from 0 to 100 points. Higher scores indicate greater disability [37]. The NPAD has shown to be a reliable and valid measure of disability in different languages [4, 16, 18, 19, 21, 22, 38].

The NDI consists of ten items [35]. Each item has six different assertions expressing progressive levels of pain or limitation in activities. Item scores range from 0 (no pain or limitation) to 5 (as much pain as possible or maximal limitation). The total NDI score ranges from 0 to 5 points. Higher scores indicate greater disability [35]. The NDI has shown to be a reliable and valid measure of disability in different languages [6, 7, 18–22, 29, 36, 38, 40].

For the GPE patients were asked to rate their overall perception of change since beginning treatment ranging from 3 (completely recovered) to zero (no change) to −3 (worse than ever). The reliability of the GPE was moderate to good in patients with neck pain and chronic arthritis [14, 24] and the validity was fair to moderate in patients with neck pain [6, 7, 24, 29, 40].

### Data analyses and interpretation

We dichotomized patients into two groups based on GPE scores. Patients were considered improved when they scored completely recovered (3) or much recovered (2) and stable when they scored slightly recovered (1) no change (0) or slightly worsened (−1). Baseline (*T*_0_ and *T*_1_) variables were compared between these groups using *t* tests for independent samples and Chi-square tests for categorical data.

Relevant change was analyzed by calculating the MDC and MIC. MDC was calculated as 1.96 × √2 ×  standard error of measurement (SEM). The SEM was calculated in stable patients as SD × √(1 − *r*) where *r* is the test–retest reliability coefficient expressed in ICC value and SD is the standard deviation of the baseline scores [30, 34].

ROC curves were constructed to determine MIC for NPAD and NDI [11, 30]. The ROC cut-off point was calculated by identifying the point on the ROC curve nearest to the upper left-hand corner, which is considered to be the best cut-off for which the sum of the percentages of false positives and false negatives classifications ([1 − sensitivity] + [1 − specificity]) is smallest [11].

Responsiveness was assessed by examining areas under ROC-curve (AUC) and correlations between change scores of NPAD and NDI, and GPE. AUC was obtained to describe the ability of the NPAD and NDI to distinguish improved patients from stable patients [30]. AUC of 0.50 indicates the questionnaire has no diagnostic accuracy beyond chance, whereas a value of 1.00 would indicate perfect accuracy [30]. AUC of at least 0.70 was considered adequate [34].

A visual method called ‘anchor-based MIC distribution’ [11] method was used to integrate anchor-based and distribution-based approaches. For the improved and stable group the distribution of the change scores on the NPAD and NDI were depicted in a graph [11, 12]. All statistical analyses were performed with SPSS software, version 18.0. The critical value for significance was *p* < 0.05.

## Results

During the recruitment period 391 patients with CNP were referred to the Center for Rehabilitation. A total of 129 patients, of which 4 were with status post neck surgery, were admitted for multidisciplinary outpatient rehabilitation. A total of 125 patients fulfilled inclusion criteria for this study. During the waiting period 14 patients decided not to start with the rehabilitation program because of practical reasons unrelated to the study. After the start of the rehabilitation program 35 patients decided not to continue because of lack of further interest or practical reasons. A dataset of 76 patients who completed the program was collected. The clinical characteristics of these patients and of the 35 dropouts are presented in Table 1. After the rehabilitation program 6 patients were completely recovered as assessed with GPE, 39 much recovered, 17 slightly recovered, 10 no change, 3 slightly worsened, 0 much worsened and 1 worse than ever. In total 45 (60 %) patients were labeled as improved and 30 (40 %) patients as stable. Baseline differences between improved and stable patients were non-significant, as were baseline differences between improved and stable patients on the one hand and dropouts on the other hand. More male patients dropped out than female patients.

**Table 1:** Baseline characteristics of improved and stable patients, and dropouts

	Improved patients (*n* = 45)	Stable patients (*n* = 30)	Dropouts (*n* = 35)
Age (years)	37.7 (12.3)	39.5 (12.0)	39.2 (10.1)
Duration of chronic pain (months)	18.5 (9.3–58.5)^a^	24.0 (9.0–69.0)^a^	18.0 (7.5–48.0)^a^
Sick leave in the past year (weeks)	18.5 (19.4)	16.1 (17.6)	17.5 (20.6)
NDI (0–50)	21 (5.5)	21 (8.1)	23 (8.6)
NPAD (0–100)	50 (12.3)	53 (16.5)	56 (20.0)
VAS_pain_ (0–100)	52 (20.1)	52 (18.6)	54 (24.8)
Female (%)	67	77	49
Pain radiating to (%)			
Shoulder(s)	82	83	86
Upper arm(s)	51	40	54
Forearm(s)	33	20	46
Hand/fingers	27	17	40
Between shoulder blades	47	53	50
Pins and needles below elbow (%)	36	33	38
Concomitant complaints (%)			
Headache	84	63	71
Dizziness	36	27	35
Concentration problems	29	17	12
Nausea	11	13	12
Fatigue	69	53	62
Low back pain	33	31	49
Self reported cause of neck pain (%)			
Motor vehicle accident	56	40	37
Other trauma	9	13	17
Spontaneously/unknown	7	7	9
Stress	4	7	6
Work related	9	10	14
Other	16	23	17
Previous treatment for neck pain (%)	91	93	94
Education			
Low	7	0	0
Intermediate	69	79	82
High	24	21	18
Work status (self employed/employee) (%)	4/78	10/80	17/60
Involved in litigation (%)	40	27	29

Values are means (SD) unless otherwise indicated

*NPAD* Neck Pain and Disability Scale, *NDI* Neck Disability Index, *VAS* Visual Analog Scale

^a^Median and interquartile range for duration of pain (months)

The results for NPAD and NDI at baseline and follow-up and the change scores in the stable and improved groups are shown in Table 2. The ICC values calculated for stable patients were 0.52 (95 % CI 0.33–0.67) for NPAD and 0.86 (95 % CI 0.79–0.91) for NDI. The SEM values of the stable patients were 11.4 for NPAD and 3.0 for NDI. These values resulted in MDC values of 31.7 points for NPAD and 8.4 for NDI.

**Table 2:** Baseline, follow-up and mean change scores of NPAD and NDI for the total, improved, and stable group of patients

	Baseline mean (SD)	Follow-up mean (SD)	Change mean (SD)	95 % CI	*p* value
NPAD					
Total (*n* = 76)^a^	51 (14.0)	36 (19.0)	15 (17.4)	11.1–19.2	<0.001
Improved (*n* = 45)	50 (12.3)	29 (17.3)	21 (16.1)	15.7–25.7	<0.001
Stable (*n* = 30)	53 (16.5)	46 (16.9)	7 (16.2)	0.3–13.1	0.04
NDI					
Total (*n* = 76)	21 (6.6)	15 (7.2)	6 (5.9)	4.2–7.0	<0.001
Improved (*n* = 45)	21 (5.5)	13 (6.2)	8 (6.3)	5.7–9.6	<0.001
Stable (*n* = 30)	21 (8.1)	18 (7.7)	3 (4.2)	1.2–4.4	0.001

*NPAD* Neck Pain and Disability Scale, *NDI* Neck Disability Index

^a^One patient scored ‘worse than ever’ and was not included in the improved or stable group, but was included in the total group

**Fig. 1:**
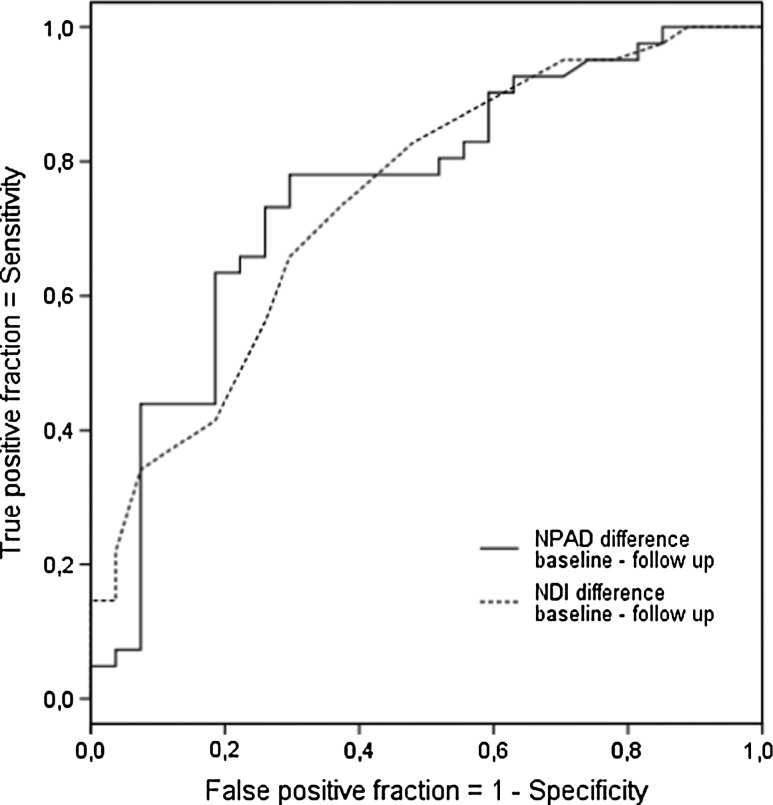
Receiver operator characteristic (ROC) curves of NPAD and NDI change scores

ROC curves for NPAD and NDI are presented in Fig. 1. The ROC cut-off MIC was for NPAD 11.5 points (sensitivity 0.74; specificity 0.70) and for NDI 3.5 points (sensitivity 0.74; specificity 0.66). Changes should exceed these values of MDC and MIC cut-offs (31.7 and 11.5 for NPAD and 8.4 and 3.5 for NDI) to be interpreted as relevant. The AUC for NPAD was 0.75 (95 % CI 0.62–0.87) and for NDI 0.75 (95 % CI 0.64–0.87). The correlation between change scores of NPAD and NDI, and GPE were, respectively, 0.48 (95 % CI 0.29–0.64) and 0.49 (95 % CI 0.30–0.64).

**Fig. 2:**
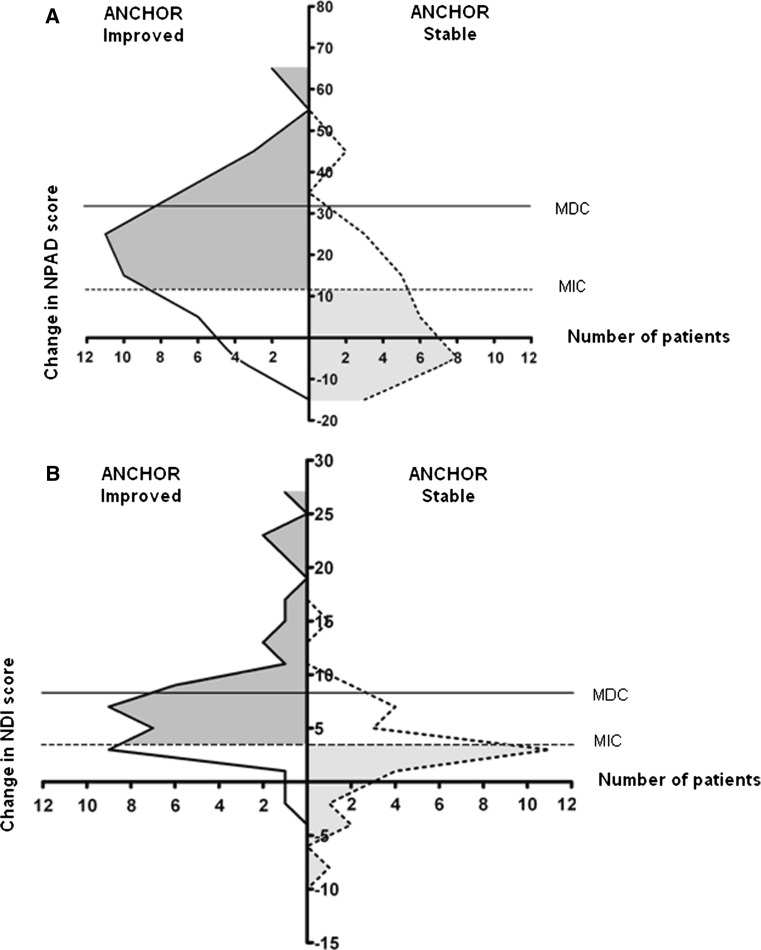
Distribution of NPAD-change scores in anchor-based improved and stable patients with indication of MIC at 11.5. At this point sensitivity = 0.74 and specificity = 0.70 (**a**). Distribution of NDI-change scores in anchor-based improved and stable patients with indication of MIC at 3.5. At this point sensitivity = 0.74 and specificity = 0.66 (**b**). *Solid curve* represents improved patients and *dotted curve* represents stable patients. The *gray parts* of the improved and stable patients represent the true positives (*dark gray*) and the true negatives (*light gray*), respectively. MDC is indicated at 31.7 for NPAD and at 8.4 for NDI

The ‘anchor-based MIC distribution’ graphs for NPAD and NDI are presented in Fig. 2a and b. These figures illustrate the effect of using MIC cut-off for change scores in the distribution of true and false positives and negatives. When the change score equals MIC, 26 % of the anchor-based improved patients have a lower change score. They are considered false negatives because the sensitivity of NPAD and NDI = 0.74. When the change score equals MIC, 30 % (NPAD) and 34 % (NDI) of the anchor-based stable patients have higher change scores. They are considered false positives because specificity of NPAD = 0.70 and of NDI = 0.66.

## Discussion

This study demonstrated that relevant change on both NPAD and NDI assessed with MDC or MIC resulted in different cut-offs with different amounts of certainty that the patient is improved. Furthermore, it demonstrated that the responsiveness of NPAD and NDI was similar when using the AUCs and the correlations between change scores and the GPE.

There is no consensus regarding the number of SEMs required to express statistically clinically relevant change: 1 × SEM, 1.65 × SEM or 1.9 × SEM. We used the 1.96 × SEM to correspond with 95 % CI. In the present study MDC for NPAD and NDI was 31.7 and 8.4, respectively. In a previous NPAD study [4] [mean baseline score 39.8 (SD 23.3)] the ICC was 0.97, the SEM 3.8 scale points, and follow up 1–2 weeks; therefore, the MDC of 10.5 was low compared with the present study. The ICCs of 0.52 for NPAD and 0.86 for NDI in the present study measured on “stable patients” was compared with the ICCs of 0.76 for NPAD and 0.84 for NDI in a previous study in the same setting with a retest interval of 18 days [18]. Larger instability of the NPAD may be explained by differences in operationalizations of neck disability between items of the NPAD and the NDI [35, 37]. Post hoc analysis showed that the amount of variation of the NPAD could be attributed to significant differences in seven individual items (2, 6, 8–12) of the questionnaire. With an ICC of 0.76 for NPAD the MDC would be 22.4. Previous NDI studies report for MDC ranges between 1.7 and 13.4 [5–7, 29, 33, 36, 39, 40]. Apart from different patient populations, the observed differences are most likely the result of different formula for the MDC calculation (1.96 or 1.65 × √2 ×  SEM) and large ranges in SEM (0.60–4.4) in these NDI studies [5–7, 29, 33, 36, 39, 40].

MIC is defined as “the smallest change that is important to patients” [8, 10, 17, 33, 34]. How to classify the smallest important change and patients as improved or stable with GPE scale levels, is an arbitrary decision [5–7, 10, 29, 36, 38–40]. In most studies using GPE as external standard, a 15-point scale was used with ≥3 (moderately better) as cut-off to distinguish improved from stable patients [6, 7, 29, 39, 40]. We classified patients as improved when their score completely recovered or much recovered to reflect important improvement similar to other studies [11, 29]. Consequently, this may lead to overestimation of the MIC. In the present study the MICs for NPAD and NDI were 11.5 and 3.5. No values of MIC for NPAD have been reported by others. MIC for NDI has been reported to range from 3.5 to 9.5 [5–7, 29, 33, 39, 40]. Differences between these studies and the present study could be the result of several factors, such as different external criteria (prognostic estimate of change [33], Health Transition Item of SF-36 [5] and GPE by patient [6, 7, 29] or by patient and therapist [39]), the number of scale levels of the external criteria, the combination of scale levels to form the improved and stable group, characteristics of population (such as age, nature and acuity of neck condition, patient setting, baseline scores), treatment, and period of follow up [5–7, 29, 39, 40].

The AUC was used to determine the probability that the improved patient can be correctly distinguished from the stable patient. In this study NPAD and NDI both have an AUC of 0.75 which is a satisfactory result and in line with results found by other studies (range 0.57–0.90) [6, 7, 22, 29, 32, 33, 39, 40]. The AUC of 0.90 for the NDI was reported in a study using a prognostic estimate of change as external criterion made by clinicians at patient’s initial visit [33]. In one study [22] responsiveness of NPAD and NDI was also compared using AUC. This study reported an AUC of 0.79 for both NPAD and NDI.

Clinicians should be aware of the fact that choosing either the MDC or the MIC cut-off gives different values and amounts of certainty on whether the observed change is relevant. Smaller values for the MIC were observed in almost all neck pain studies including the present study [5–7, 29, 39, 40]. Using the anchor-based MIC the proportion of false positives and false negatives is found to be the smallest. By raising the cut-off, the probability of false positives is reduced and the probability of false negatives is increased [12]. Applying the more conservative MDC, the certainty that the change score is relevant and larger than the measurement error, is high. The amount of certainty needed may depend on the consequences in patient care and could be a case by case decision.

For example for risk, full neck surgery or an expensive time-consuming multidisciplinary rehabilitation the more conservative MDC cut-off could be used, while in primary care setting the more liberal MIC cut-off could be used. On the other hand, socio-economic factors such as chance of returning to work as result of a therapy may be also of importance as external criterion for relevant change.

In the present study, the visual ‘anchor-based MIC distribution’ method was used whereby the distribution of the change scores on the NPAD and NDI was depicted in curves. The narrower the curves and the smaller the overlap of the curves, the smaller the chance of misclassification [12]. Both aspects of the curves largely depended on the correlation between change scores of NPAD and NDI and GPE as anchor [12]. In the present study these correlations were similar to those of most other NPAD (range 0.42–0.59) [16, 22, 38] and NDI (range 0.19–0.58) studies [6, 7, 22, 32, 38–40]. The GPE as external criterion to operationalize relevant change has been criticized because it consists of only one question and patient’s ability to recall their previous health status is questionable [15, 27]. Any anchor-based approach is as good as the used external criterion and the methodology to define relevant change.

The present study is conducted in a university setting and is therefore representative of patients with CNP in a tertiary referral center. Percentage of females was similar to that of other responsiveness studies [4–7, 16, 21, 22, 29, 32, 33, 36, 38, 40]. Mean age in our study was lower (38.5 years) compared with other responsiveness studies [4–7, 16, 21, 22, 29, 32, 33, 36, 38, 40]. A potential limitation of this study is that the sample consisted largely of patients with moderate neck pain and disability. Although this may be expected in this tertiary rehabilitation setting generalizability beyond this setting cannot be assumed. The dropouts did not introduce bias because this study was aimed to measure the questionnaires and not the effect of the rehabilitation program. The strength of this study is that relevant changes were assessed with MDC and MIC on the NPAD and NDI and that a head-to-head comparison of the responsiveness of NPAD and NDI was performed. Further study of MDC, MIC, and responsiveness of NPAD and NDI is necessary to assess the measurement properties in other patient groups and also in comparison with other external criteria for relevant change.

## Conclusion

Relevant change of both NPAD and NDI assessed with MDC and MIC resulted in different cut-offs with different amounts of certainty that the patient is improved. Responsiveness of NPAD and NDI was similar.
